# Timing of Antiretroviral Therapy and Systemic Inflammation in Sub-Saharan Africa: Results From the META Longitudinal Cohort Study

**DOI:** 10.1093/infdis/jiz259

**Published:** 2019-06-12

**Authors:** Mark J Siedner, Mwebesa Bosco Bwana, Stephen Asiimwe, Gideon Amanyire, Nicholas Musinguzi, Jose Castillo-Mancilla, Russell P Tracy, Ingrid T Katz, David R Bangsberg, Peter W Hunt, Catherine Orrell, Jessica E Haberer, META study investigators, Norma Ware, Tumwesigye Elioda, Alexander C Tsai, Lynn Matthews, Monique Wyatt

**Affiliations:** 1 Harvard Medical School, Boston; 2 Massachusetts General Hospital, Boston; 3 Mbarara University of Science and Technology, Uganda; 4 Africa Health Research Institute, Kwa-Zulu Natal, South Africa; 5 Kabwohe Clinical Research Center, Uganda; 6 University of Colorado, Denver; 7 University of Vermont, Burlington; 8 Brigham and Women’s Hospital, Boston; 9 Oregon Health & Science University-Portland State University School of Public Health, Portland; 10 University of California, San Francisco; 11 University of Cape Town, South Africa

**Keywords:** HIV, inflammation, immune activation, antiretroviral therapy, Uganda, South Africa

## Abstract

Chronic inflammation predicts complications in persons with human immunodeficiency virus infection. We compared D-dimer, soluble CD14, and interleukin 6 levels before and 12 months after antiretroviral therapy (ART) initiation, among individuals starting ART during earlier-stage (CD4 T-cell count >350/µL) or late-stage disease (CD4 T-cell count <200/µL). Female sex, older age, viral load, and late-stage disease were associated with pre-ART biomarkers (n = 661; *P* < .05). However, there were no differences in biomarkers by disease stage after 12 months of ART (n = 438; *P* > .05), owing to loss from observation and greater declines in biomarkers in late-stage initiators (*P* < .001). Earlier initiation of ART is associated with decreased inflammation, but levels seem to converge between earlier and later initiators surviving to 12 months.

Early initiation of antiretroviral therapy (ART) in sub-Saharan Africa results in improved immune reconstitution, reductions in biomarkers of inflammation, and reductions in AIDS events [1]. However, it is less certain how the timing of ART initiation affects the degree of chronic inflammation among persons living with human immunodeficiency virus (HIV) in the region [2]. Even less is known about how the timing of ART affects chronic HIV complications in sub-Saharan Africa, where individuals continue to present to care with relatively advanced disease [3].

We leveraged a multinational cohort study in sub-Saharan Africa with longitudinal observation of individuals initiating ART at earlier-stage versus late-stage disease to understand how ART timing affects biomarkers of immune activation before and 12 months after ART initiation. We hypothesized that individuals starting ART during late-stage disease would have higher levels of biomarkers before treatment and that this difference would not be resolved after 12 months of suppressive therapy.

## METHODS

### Study Setting and Participants

The Monitoring of Early Treatment Adherence (META) study was an observational longitudinal cohort study designed to discern how timing of ART initiation and pregnancy affect ART adherence in Uganda and South Africa. Full protocol details have been published elsewhere [4]. For this analysis, we included individuals in the late-stage disease (nonpregnant with a CD4 T-cell count <200/µL) and earlier-stage disease (nonpregnant with a CD4 T-cell count >350/µL and World Health Organization stage I) subgroups. Participants were observed at the start of treatment and again at 6 and 12 months for completion of surveys and blood collection.

### Laboratory Methods

CD4 T-cell counts were measured using the PIMA assay (Alere Diagnostics). HIV-1 RNA viral load (VL) was measured with the Cobas Taqman platform in Uganda and the Roche CAP/CTM HIV-1 v2 platform in South Africa. We defined viral suppression as <39 copies/mL in South Africa and <33 copies/mL in Uganda. We excluded participants with viral suppression at enrollment. Blood at each visit was collected into ethylenediaminetetraacetic acid tubes, immediately centrifuged for plasma separation, and stored at −80^o^C. We tested cryopreserved plasma for 3 biomarkers: (1) interleukin 6 (IL-6) (MesoScale Discovery); (2) soluble CD14 (sCD14; R&D Systems); and (3) D-dimer (Diagnostica Stago). We imputed the minimum and maximum limits of quantification for each assay for results out of range (500 and 3500 ng/mL for sCD14, 0.01 and 20 μg/µL for D-dimer). No participants had out-of-range IL-6 levels. Biomarkers were selected for their association with HIV infection, and because each has been predictive of long-term chronic comorbid conditions and/or death [5–7]. Biomarkers were tested at the University of Vermont Laboratory for Clinical Biochemistry Research.

### Statistical Methods

We fit 3 sets of linear regression models with the following outcomes of interest: (1) each biomarker before ART initiation; (2) each biomarker after 12 months of ART, restricted to those with 12-month viral; and (3) changes in each biomarker from before treatment to 12 months, restricted to those with 12-month viral suppression. Biomarkers were log-transformed and divided by their interquartile range (IQR), such that each 1-unit increase in the coefficient represents an increase in log-transformed biomarker IQR [8, 9].

Our primary explanatory variable was earlier- versus late-stage disease subgroup. Models were fit with and without potential confounders, including age (10-year increments), country, sex, current smoking, body mass index, and pretreatment VL (log_10_). We graphically depicted adjusted changes in biomarkers over time and by subgroup using postregression margins. To assess the potential bias due to loss from observation and death before 12 months, we compared pretreatment biomarkers between those with and without inflammatory data at 12 months. In secondary analyses, we repeated the models of change in biomarkers with a sex-by-subgroup, age-by-subgroup (using the median cohort age of 33 years), or a country-by-subgroup interaction term. Finally, we repeated models after removing pretreatment VL as a confounder and without restricting the cohort to those who achieved 12-month viral suppression.

### Ethical Considerations

Study procedures were approved by Partners Healthcare, the Mbarara University of Science and Technology, Uganda National Council for Science and Technology, University of Cape Town, and Western Cape province in South Africa. All participants provided written informed consent.

## RESULTS

We enrolled 699 individuals in the earlier-stage (n = 335) or late-stage (n = 364) disease groups (Supplementary Figure 1). Of these, 661 (95%) had pretreatment biomarker testing, had a detectable VL at enrollment, and were included in our pretreatment analyses. Our primary 12-month analytic data set included 438 individuals (79%) who completed 12-month biomarker testing and achieved virologic suppression at 12 months. Of these, 113 were excluded from the 12-month primary analysis for a detectable VL at study conclusion. Another 110 were excluded from 12-month analyses, with the most common reasons being death (n = 34) and lack of plasma for biomarker testing (n = 43).

The median age of the cohort was 33 years, approximately 60% were female. and the study was approximately evenly divided between participants from Uganda and South Africa (Supplementary Table 1A and 1B). The median CD4 T-cell counts in the earlier- and late-stage subgroups were 427/µL and 118/µL, respectively. All participants were started on a regimen of efavirenz, tenofovir, and emtricitabine. Individuals in the earlier-stage group were more likely to be female and had lower pretreatment VL (*P* < .001).

Individuals starting ART during earlier-stage disease had lower levels of all 3 biomarkers before ART treatment than the late-stage group (*P* < .001; Table 1 and Supplementary Figure 2). These relationships were persistent after adjustment for confounders, such that initiating ART with late-stage disease was associated with a mean increase of 0.5–1.5 times the IQR for each biomarker. In all models, pretreatment VL was associated with increased levels of biomarkers. In adjusted models, older individuals had higher pretreatment levels of D-dimer and IL-6, women had higher levels of D-dimer, and participants in South Africa had lower levels of sCD14 than those in Uganda.

**Table 1.** Association Between Pretreatment Biomarkers of Inflammation and Earlier/Asymptomatic Versus Late-Stage Disease at Antiretroviral Therapy Initiation, With and Without Adjustment for Confounding Factors (n = 661)^a^

Factor	Soluble CD14				IL-6				D-Dimer			
	Univariable		Multivariable		Univariable		Multivariable		Univariable		Multivariable	
	Estimate (95% % CI)	*P* Value	Estimate (95% CI)	*P* Value	Estimate (95% CI)	*P* Value	Estimate (95% CI)	*P* Value	Estimate (95% CI)	*P* Value	Estimate (95% CI)	*P* Value
Age (10-y increments)	0.03 (−.04 to .10)	.41	0.07 (.01–.13)	.03	0.25 (.05–.46)	.02	0.27 (.08–.45)	.01	0.27 (.07–.47)	.01	0.32 (.13–.51)	.001
Female sex	−0.11 (−.25 to .02)	.10	0.16 (.03–.29)	.02	−0.33 (−.73 to −.08)	.11	0.26 (−.14 to .66)	.20	0.34 (−.06 to .74)	.09	0.99 (.58–1.39)	<.001
BMI	−0.04 (−.05 to −.03)	<.001	−0.03 (−.04 to −.01)	<.001	−0.05 (−.09 to −.02)	.003	−0.03 (−.06 to .01)	.17	−0.04 (−.07 to .001)	.04	−0.04 (−.07 to .001)	.06
South Africa (vs Uganda)	−0.18 (−.31 to −.04)	.01	−0.21 (−.33 to −.08)	.001	0.46 (.07–.86)	.02	0.28 (−.10 to .67)	.15	0.53 (.14–.92)	.01	0.27 (−.12 to .67)	.17
Current smoking status	−0.06 (−.29 to .17)	.61	−0.21 (−.25 to .16)	.70	0.25 (−.42 to .92)	.47	0.02 (−.61 to .65)	.95	0.18 (−.48 to .84)	.60	0.19 (.44–.83)	.55
Pretreatment VL (log_10_)	0.37 (.31–.44)	<.001	0.25 (.18–.32)	<.001	0.95 (.75–1.14)	<.001	0.55 (.34–.76)	<.001	0.89 (.70–1.08)	<.001	0.63 (.41–.84)	<.001
Late ART (vs earlier ART)	0.38 (.31–.44)	<.001	0.49 (.35–.62)	<.001	1.07 (.89–1.25)	<.001	1.63 (1.23–2.03)	<.001	0.87 (.68–1.05)	<.001	1.26 (.86–1.67)	<.001

Abbreviations: ART, antiretroviral therapy; BMI, body mass index; CI, confidence interval; IL-6, interleukin 6; VL, human immunodeficiency virus type 1 RNA viral load.

^a^Estimates represent values for biomarkers of inflammation log-transformed and divided by the interquartile range (IQR) of the distribution, such that the coefficient represents a 1-unit change in the IQR for that biomarker.

**Figure 1.**
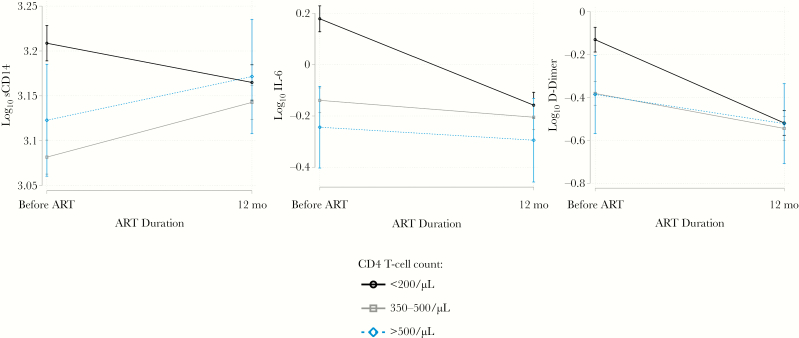
Log transformed biomarkers of inflammation prior to and 12 months after antiretroviral therapy among those achieving virologic suppression at 12 months (n = 438).

When comparing pretreatment biomarkers between patients who were retained in care and underwent testing at 12 months (n = 438) and those lost to observation or with detectable VL at 12 months (n = 223), we found higher levels of pretreatment biomarkers among those who were lost to observation in the late-stage group (all *P* < .01; Supplementary Figure 3). This association was not observed among those in the earlier-stage group. Among participants who survived and achieved viral suppression at 12 months, we found no differences in levels of any biomarker between those in the earlier-and late-stage groups (P > .05; Table 2 and Figure 1).

**Table 2.** Association Between 12-Month Biomarkers of Inflammation and Earlier/Asymptomatic Versus Late-Stage Disease at Antiretroviral Therapy Initiation, With or Without Adjustment for Confounding Factors (n = 438)^a^

Factor	Soluble CD14				IL-6				D-Dimer			
	Univariable		Multivariable		Univariable		Multivariable		Univariable		Multivariable	
	Estimate (95% CI)	*P* Value	Estimate (95% CI)	*P* Value	Estimate (95% CI)	*P* Value	Estimate (95% CI)	*P* Value	Estimate (95% CI)	*P* Value	Estimate (95% CI)	*P* Value
Age (10-y increments)	−0.04 (−.12 to .04)	.32	−0.01 (−.10 to .07)	.74	0.22 (.05–.40)	.01	0.14 (−0.04 to .31)	.13	0.08 (−.13 to .30)	.45	0.12 (−.09 to .34)	.27
Female sex	0.03 (−.13 to .19)	.73	0.17 (−.01 to .34)	.06	0.09 (−.28 to .46)	.63	0.10 (−0.29 to .48)	.61	1.20 (.77–1.63)	<.001	1.32 (.84–1.80)	<.001
BMI	−0.02 (−.04 to −.01)	.001	−0.01 (−.03 to .004)	.14	0.04 (.01–.07)	.01	0.02 (−.01 to .06)	.14	0.04 (−.00 to .08)	.05	0.0005 (−.04 to .05)	.98
South Africa (vs Uganda)	−0.35 (−.51 to −.20)	<.001	−0.38 (−.55 to −.21)	<.001	1.15 (.81–1.48)	<.001	0.92 (.55–1.30)	<.001	0.46 (.03–.88)	.04	0.31 (−.15 to .78)	.19
Current smoking status	0.12 (−.13 to .36)	.35	0.27 (.01–.52)	.04	0.43 (−.13 to .98)	.13	0.04 (−.53 to .60)	.89	−0.19 (−.86 to .49)	.59	0.10 (−.60 to .81)	.78
Pretreatment VL (log_10_)	0.09 (.01–.17)	.03	0.11 (.02–.20)	.02	0.36 (.18–.54)	<.001	0.35 (.15–.54)	.001	0.19 (−.03 to .40)	.09	0.27 (.02–.51)	.03
Late ART (vs earlier ART)	0.05 (−.03 to .13)	.21	−0.02 (−0.20 to .15)	.79	0.13 (−.05 to .31)	.15	0.09 (−.29 to .47)	.65	0.05 (−.16 to .27)	.62	0.10 (−.38 to .57)	.70

Abbreviations: ART, antiretroviral therapy; BMI, body mass index; CI, confidence interval; IL-6, interleukin 6; VL, human immunodeficiency virus type 1 RNA viral load.

^a^Estimates represent values for biomarkers of inflammation log-transformed and divided by the interquartile range (IQR) of the distribution, such that the coefficient represents a 1-unit change in the IQR for that biomarker.

The equivalence between subgroups at 12 months was explained by greater decreases in each of the biomarkers from before treatment to 12 months among individuals in the late-stage subgroup (*P* < .003 for all biomarkers; Table 3). Female sex (D-dimer), active smoking (sCD14), and a higher pretreatment VL (sCD14, IL-6, and D-dimer) were associated with higher biomarker levels at 12 months. The relationship with country of origin was inconsistent, with South Africans having lower levels of sCD14, but higher levels of IL-6. We found no meaningful differences in these relationships in sensitivity analyses, including those with a detectable VL at 12 months (Supplementary Table 2) and without inclusion of pretreatment VL (Supplementary Table 3). We found similar relationships between disease stage at ART initiation and changes in biomarkers from before treatment to 12 months in analyses stratified by sex, country of origin, and age (Supplementary Figure 4).

**Table 3.** Association Between Change in Biomarkers of Inflammation From Before Treatment to 12 Months and Earlier/Asymptomatic Versus Late-Stage Disease at Antiretroviral Therapy Initiation, With or Without Adjustment for Confounding Factors (n = 438)^a^

Factor	Soluble CD14				IL-6				D-Dimer			
	Univariable		Multivariable		Univariable		Multivariable		Univariable		Multivariable	
	Estimate (95% CI)	*P* Value	Estimate (95% CI)	*P* Value	Estimate (95% CI)	*P* Value	Estimate (95% CI)	*P* Value	Estimate (95% CI)	*P* Value	Estimate (95% CI)	*P* Value
Age (10-y increments)	0.07 (−.01 to .14)	.07	0.05 (−.02 to .13)	.14	−.01 (−.07 to .10)	.72	0.03 (−.05 to .11)	.49	0.07 (−.01 to .15) 0.0005	.10	0.08 (−.01 to .16)	.08
Female sex	−0.11 (−.26 to .05)	.17	−0.02 (−.18 to .14)	.81	−0.06 (−.23 to .10)	.46	0.09 (−.09 to .27)	.33	−0.13 (−.30 to .05)	.15	0.03 (−.15 to .22)	.72
BMI	−0.01 (−.02 to .01)	.25	.0001 (−.01 to .02)	.98	−0.02 (−.04 to −.01)	.004	−0.004 (−.02 to .01)	.60	−0.02 (−.03 to −.002)	.02	−0.01 (−.02 to .01)	.52
South Africa (vs Uganda)	0.19 (.04–.34)	.01	0.17 (.01 to −.33)	.03	−0.22 (−.38 to −.06)	.008	−0.25 (−.43 to −.08)	.004	−0.05 (−.22 to .11)	.54	−0.11 (−.29 to .07)	.23
Current smoking status	−0.01 (−.24 to .23)	.96	−0.17 (−.40 to .07)	.17	0.07 (−.18 to .33)	.58	0.12 (−.14 to .38)	.07	0.13 (−.13 to .39)	.34	0.08 (−.19 to .36)	.56
Pretreatment VL (log_10_)	0.24 (.16–.31)	<.001	0.13 (.05–.21)	.002	0.17 (.09–.25)	<.001	0.08 (−.01 to .18)	.07	0.19 (.11–.28)	<.001	0.12 (.03–.22)	.01
Late ART (vs earlier ART)	0.27 (.20–.34)	<.001	0.42 (.26–.58)	<.001	0.26 (.18–.34)	<.001	0.42 (.25–.60)	<.001	0.20 (.12–.29)	<.001	0.27 (.09–.46)	.004

Abbreviations: ART, antiretroviral therapy; BMI, body mass index; IL-6, interleukin 6; VL, human immunodeficiency virus type 1 RNA viral load.

^a^Estimates represent values for biomarkers of inflammation log-transformed and divided by the interquartile range (IQR) of the distribution, such that the coefficient represents a 1-unit change in the IQR for that biomarker.

## DISCUSSION

This study showed that individuals in Uganda and South Africa starting ART with late-stage disease have significantly higher levels of sCD14, IL-6, and D-dimer than those starting ART during earlier stages of disease, and it offers additional evidence of the many benefits of earlier ART initiation in the region. However, contrary to our hypothesis, among those who survive and achieve viral suppression at 12 months, we found no difference in levels of biomarkers comparing those initiating treatment with earlier- or late-stage disease 1 year after treatment initiation. This result seemed to be driven by 2 phenomena: (1) loss from care and death in those with the greatest inflammation in the late-stage group and (2) greater decreases in biomarkers in the late-stage disease group among those who survived and remained in care at 12 months. Our findings offer promise that, among survivors after late-stage disease initiation, effective ART might reduce long-term consequences of chronic inflammation in those who achieve viral suppression.

Similar to our study, individuals in both the Men’s AIDS Cohort Study (MACS) and the Women’s Interagency Health Study (WIHS) had significant reductions in most inflammatory biomarkers after ART initiation [10]. However, unlike those studies, we detected a decrease in IL-6 after ART initiation, which might be due to our use of analyte-specific enzyme-linked immunosorbent assay platforms, which are typically more sensitive to changes in biomarkers than multiplex assays. Alternatively, these differences might be because the MACS and WIHS cohorts observed individuals with relatively high CD4 T-cell counts at ART initiation compared with ours (median cohort CD4 T-cell count, approximately 300–500/µL and 187/µL, respectively). Notably, the lack of a decrease in sCD14 seen among earlier-stage initiators in our cohort has been reported elsewhere and might be due to muted reductions with efavirenz-based ART [11]. This finding argues for additional investigation, given broad use of efavirenz globally and its associations with poor outcomes [6].

Unlike studies from other settings, in this cohort from Africa, we found accentuated reductions in biomarkers in late-stage disease initiators, such that there were no difference in biomarkers in those surviving and achieving viral suppression after 12 months. In contrast, large reductions in T-cell activation, sCD14, soluble CD163, and D-dimer have been documented with earlier treatment initiation in other settings [12]. As with the WIHS and MACS cohorts, a notable contrast between these studies and ours was the relatively high CD4 T-cell counts in those studies.

An unexpected finding in our study was the difference seen in biomarkers both before and after ART between Uganda and South Africa, which varied by biomarker (eg, higher sCD14 and lower IL-6 levels in Uganda versus South Africa). Although we cannot readily explain these findings, they reinforce the need to consider region-specific relationships between HIV, ART, and these biomarkers. If on-treatment inflammation does predict future events in this region, our data support a potentially similar benefit of ART in both earlier- and late-stage disease treatment initiators who survive and attain virologic suppression. There are suggestions from Western settings that levels of inflammation after ART initiation are potentially more predictive of future complications than pretreatment levels [13]. Similarly, our group previously reported that changes in sCD14 and the ratio of kynurenine to tryptophan (KT ratio) 6 months after treatment, but not before treatment, were predictive of increased carotid intima media thickness years later in Uganda [9] and that higher levels of KT ratio, sCD14, IL-6 and D-dimer were associated with a greater risk of mortality 6 months after ART initiation [14].

Our results should also be considered generalizable to individuals in similar settings who complete 12 months of therapy and achieve virologic suppression. Notably, loss from observation occurred in approximately 20% of individuals in our analytic cohort, including excess deaths in the late ART group, which may have inflated the extent of observed declines in biomarkers. Although residual or unmeasured confounding might explain associations between treatment stage and biomarkers, our effect sizes for both pretreatment and on-treatment estimates were large enough (most *P* < .003) that a confounder of a large effect size would be needed to significantly alter our conclusions. We selected plasma soluble markers known to be associated with non-AIDS events, but other biomarkers that reflect distinct biologic pathways but also predict disease (eg, soluble tumor necrosis factor receptor and KT ratio), may behave differently. Furthermore, immune activation in lymphoid and other tissues that theoretically contribute to disease are not fully reflected by plasma biomarkers [15].

In summary, we report data from a multicenter longitudinal cohort in Africa, demonstrating that late initiation of ART is associated with increased systemic inflammation. However, in those who remain alive and achieve virologic suppression at 12 months, biomarker levels between earlier- and late-stage initiators converge at 12 months. The long-term consequences of these findings, and particularly if inflammatory biomarkers after suppressive ART continue to predict clinical outcomes in the region, is a critical area for future study.

## Supplementary Data

Supplementary materials are available at *The Journal of Infectious Diseases* online. Consisting of data provided by the authors to benefit the reader, the posted materials are not copyedited and are the sole responsibility of the authors, so questions or comments should be addressed to the corresponding author.

jiz259_suppl_Supplementary_Figure

jiz259_suppl_Supplementary-Table

## Notes

***Acknowledgments.*** We thank all study participants for their time and involvement in the study, and we thank the study staff, including the following: research assistants. Nomakhaya April, RN, Alienah Mpahleni, Vivie Situlo, Speech Mzamo, Nomsa Ngwenya, Khosi Tshangela Regina Panda, Teboho Linda, Christine Atwiine, Sheila Moonight, Edna Tindimwebwa, Nicholas Mugisha, Peace Atwogeire, Vian Namana, Catherine Kyampaire, and Gabriel Nuwagaba; program managers, Annet Kembabazi, Stephen Mugisha, Victoria Nanfuka, Anna Cross, Nicky Kelly, Daphne Moralie, and Kate Bell; statistician, N. M.; data managers, Dolphina Cogill, Justus Ashaba, Zoleka Xapa, Mathias Orimwesiga, Elly Tuhanamagyezi, and Catherine Kyampaire; laboratory managers, Don Bosco Mpanga, Leonia Kyarisima, and Simone Kigozi; and drivers, Edgar October, Silver Mugisha, and Ibrahim Kiviiri.

***META study team investigators.*** The Monitoring of Early Treatment Adherence (META) study team includes, as principal investigators, J. E. H., C. O., Norma Ware, M. B. B., S. A., G. A., Tumwesigye Elioda, and D. R. B.; and as coinvestigators, Alexander C. Tsai, M. J. S., Lynn Matthews, I. T. K., and Monique Wyatt.

***Financial support.*** This work was supported by the Bill & Melinda Gates Foundation (grant OPP113634) and by National Institutes of Health (grant K23 MH099916).

***Potential conflicts of interest.*** All authors: No reported conflicts. All authors have submitted the ICMJE Form for Disclosure of Potential Conflicts of Interest. Conflicts that the editors consider relevant to the content of the manuscript have been disclosed.
