# Supplementary material for: Timing of Antiretroviral Therapy and Systemic Inflammation in Sub-Saharan Africa: Results From the META Longitudinal Cohort Study
Source: J Infect Dis. 2019 Jun 12;220(7):1172–7. doi: 10.1093/infdis/jiz259 (PMC6736121; doi:10.1093/infdis/jiz259)

Figure 1. Biomarkers of inflammation prior to and 12 months after antiretroviral therapy among those achieving virologic suppression at 12 months (n=438)

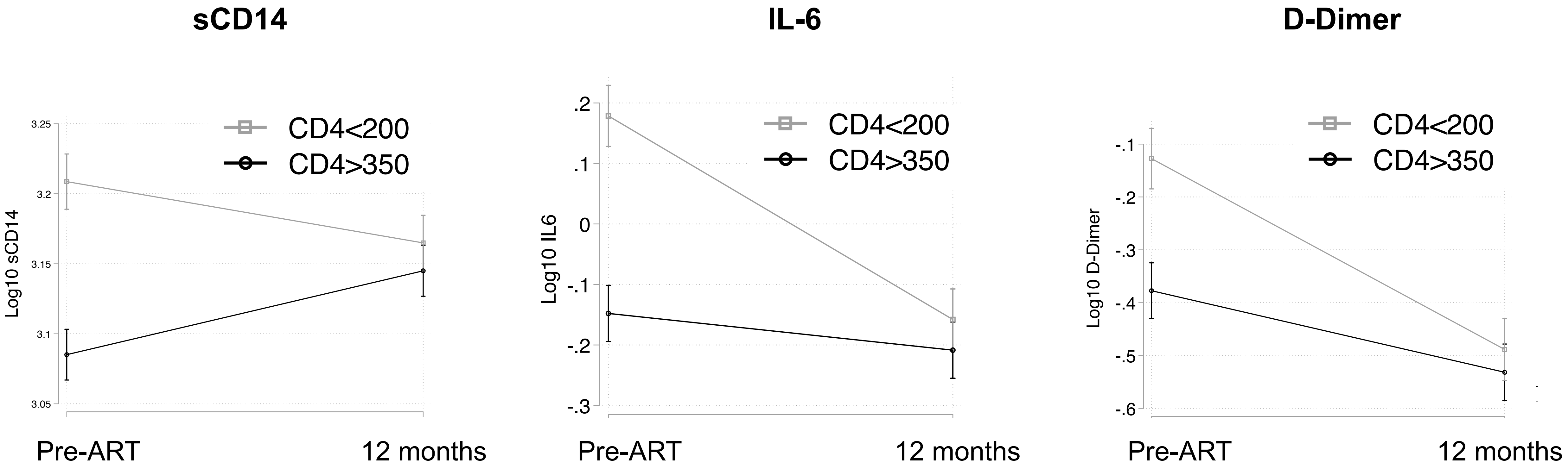

Supplemental Figure 1. Study Schema

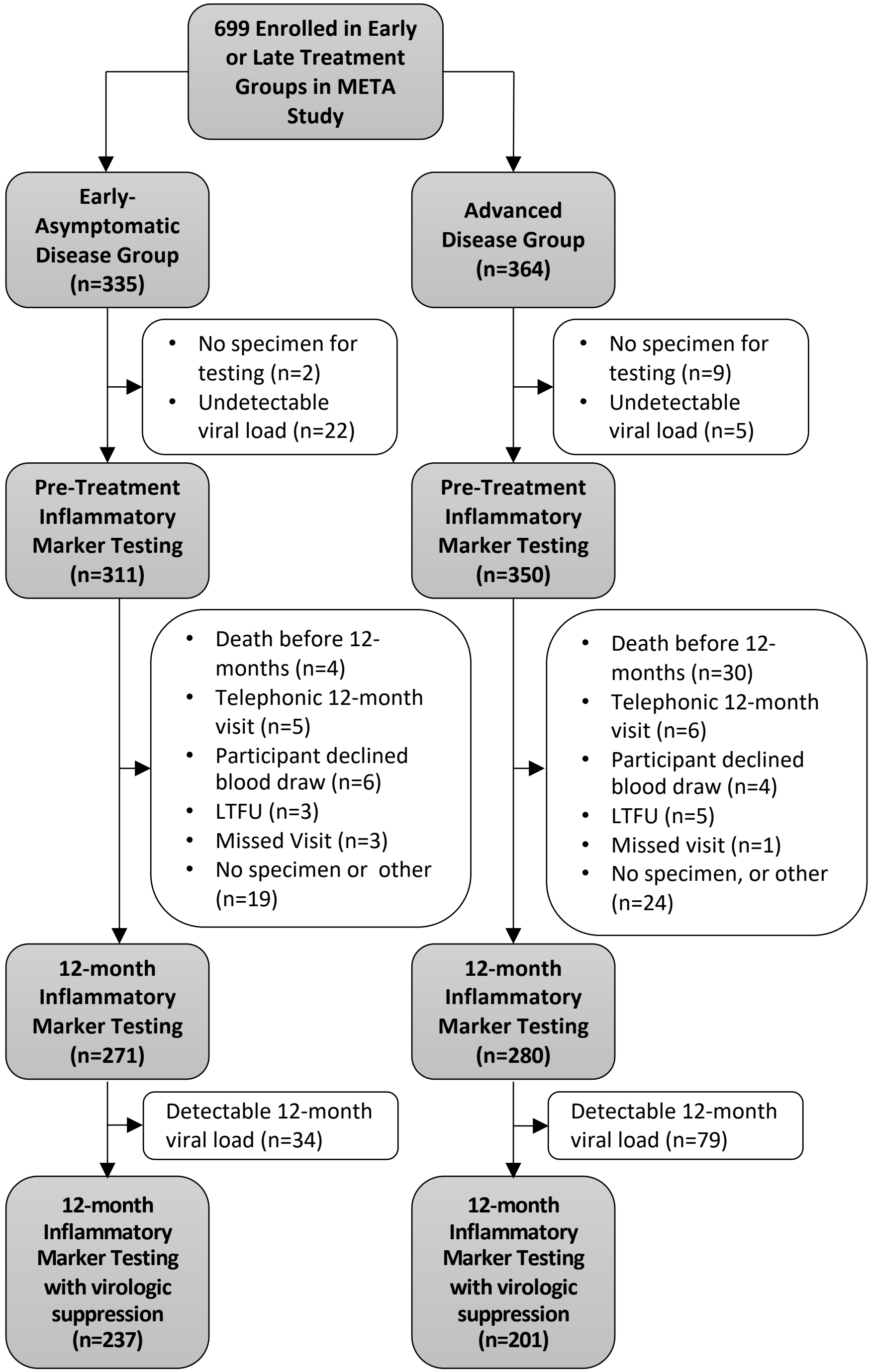

Supplemental Figure 2. Biomarkers of inflammation prior to ART initiation (n=661)

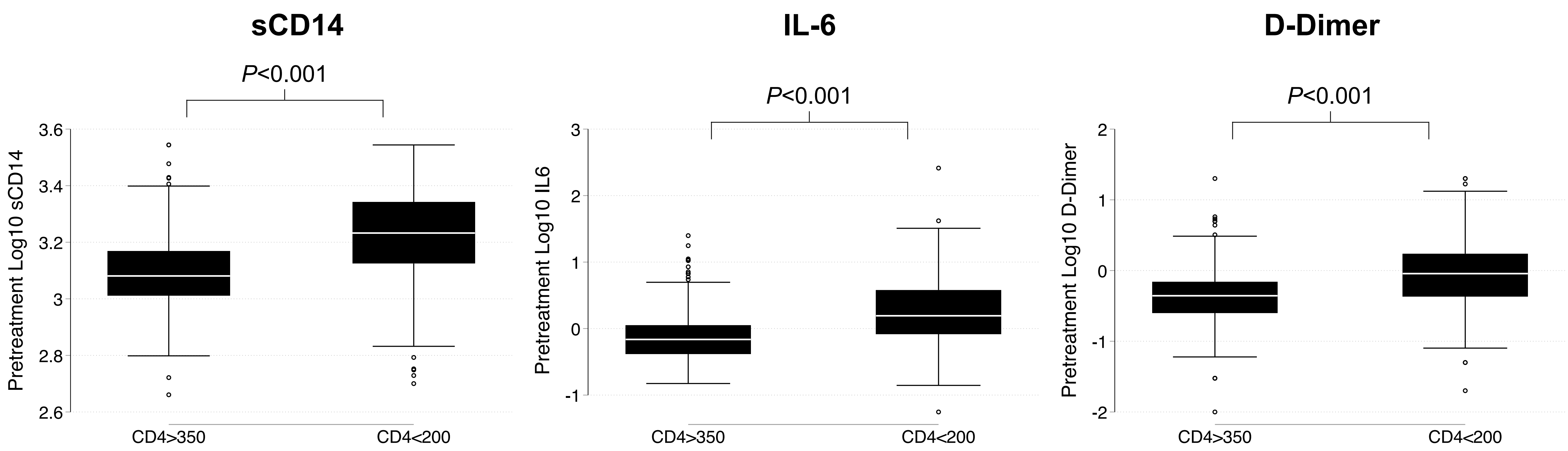

Supplemental Figure 3. Biomarkers of inflammation prior to ART initiation comparing those who completed the study and those lost from observation before 12 months

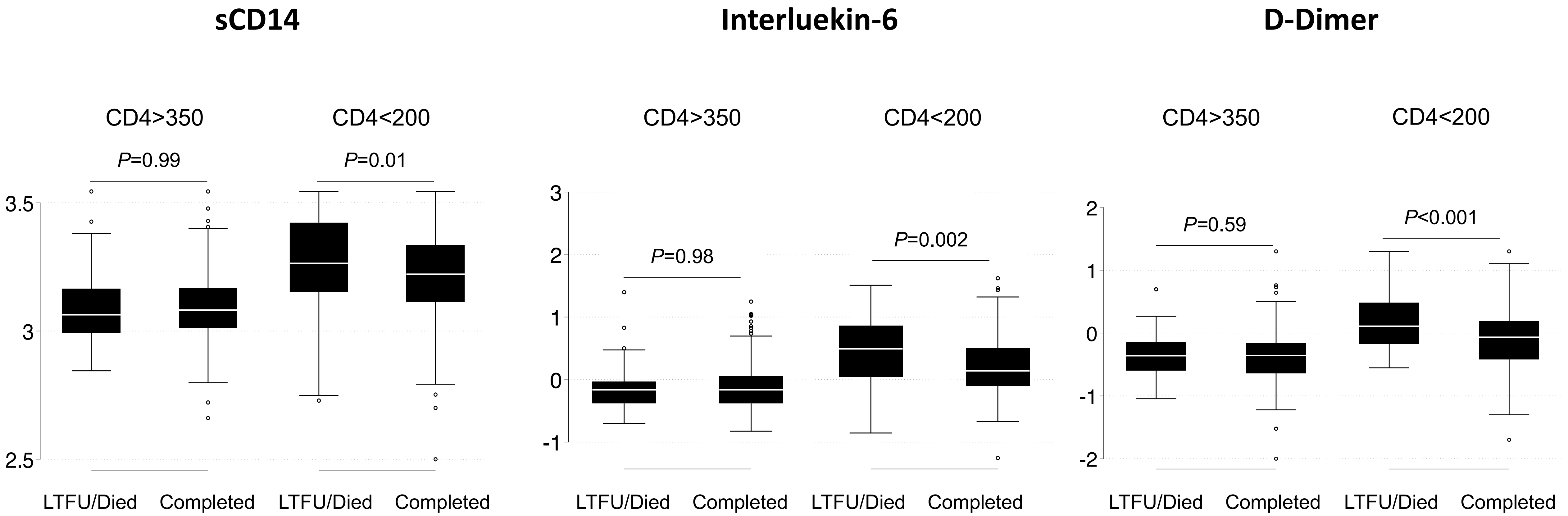

Supplemental Figure 4. Changes in biomarkers of inflammation from pre-treatment to 12 months after antiretroviral therapy initiation categorized by sex, country of origin, and age.

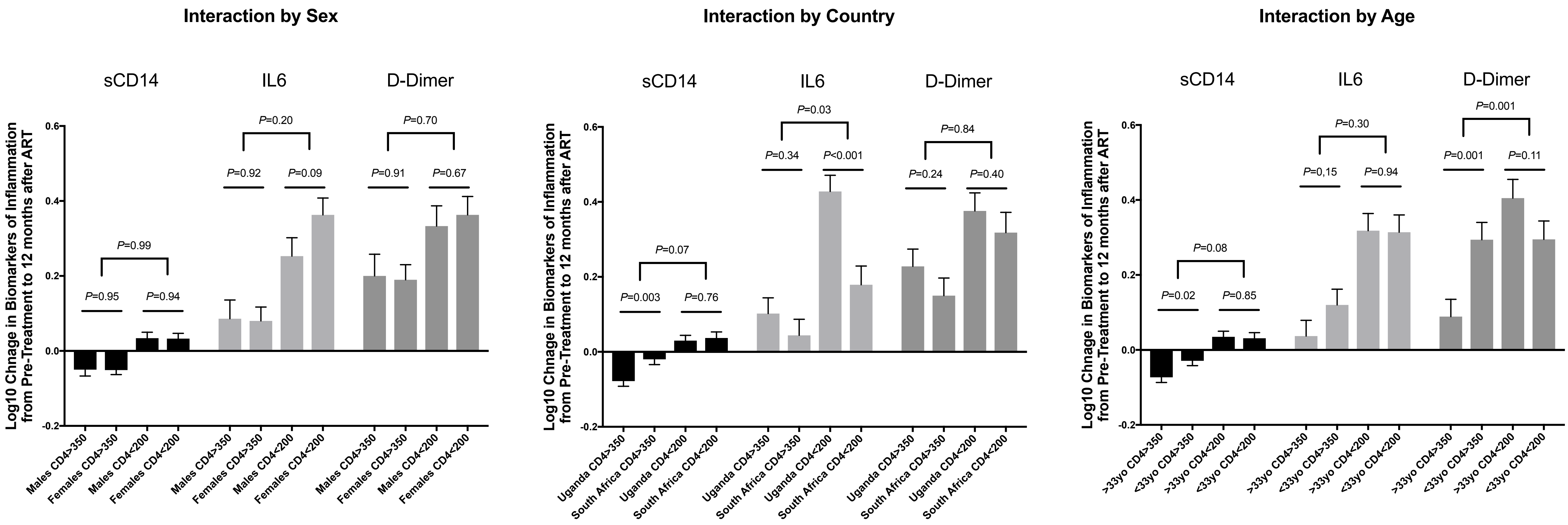

Supplement: jiz259_suppl_Supplementary_Figure [file jiz259_suppl_supplementary_figure.pdf]
