# Supplementary material for: Timing of Antiretroviral Therapy and Systemic Inflammation in Sub-Saharan Africa: Results From the META Longitudinal Cohort Study
Source: J Infect Dis. 2019 Jun 12;220(7):1172–7. doi: 10.1093/infdis/jiz259 (PMC6736121; doi:10.1093/infdis/jiz259)
Supplement: jiz259_suppl_Supplementary-Table [file jiz259_suppl_supplementary-table.docx]

Supplemental Table 1. Cohort characteristics

| **1A. Participants with pre-treatment inflammatory marker testing** | | | | |
| --- | --- | --- | --- | --- |
| Characteristic | Total Cohort  (n=661) | Earlier-Stage Group  (n=350) | Late-Stage Group (n=311) | *P*-value |
| Age (median, IQR) | 33 (27-41) | 32 (25-41) | 33 (28-40) | 0.07 |
| Female sex (n, %) | 396 (60) | 215 (69) | 181 (51) | <0.001 |
| Country (n, %) |  |  |  |  |
| Uganda | 321 (49) | 155 (50) | 166 (47) | 0.54 |
| South Africa | 340 (51) | 156 (50) | 184 (53) |  |
| Current smoker (n, %) | 64 (10) | 28 (9) | 36 (10) | 0.72 |
| Pre-treatment log10 viral load copies/mL (median, IQR) | 4.7 (4.1 – 5.2) | 4.2 (3.5 – 4.7) | 5.0 (4.6 – 5.5) | <0.001 |
| Pre-treatment CD4 count cells/μL (median, IQR) | 187 (111 - 425) | 427 (391 – 472) | 117.5 (58 – 159) | N/A |
| Soluble CD14  (median, IQR) | 1414 (1141 – 1858) | 1204 (1027 – 1474) | 1707 (1333 – 2198) | <0.001 |
| Interluekin-6  (median, IQR) | 0.96 (0.55 – 2.20) | 0.69 (0.42 – 1.13) | 1.56 (0.83 – 3.80) | <0.001 |
| D-dimer  (median, IQR) | 0.58 (0.29 – 1.20) | 0.44 (0.24 – 0.69) | 0.90 (0.42 – 1.70) | <0.001 |
| **1B. Participants with pre-treatment and 12-month inflammatory testing and with viral suppression at 12 months** | | | | |
| Characteristic | Total Cohort  (n=661) | Earlier-Stage Group  (n=350) | Late-Stage Group (n=311) | *P*-value |
| Age (median, IQR) | 33 (27 – 41) | 33 (26 – 42) | 33 (29 – 41) | 0.53 |
| Female sex (n, %) | 274 (63) | 166 (70) | 108 (54) | <0.001 |
| Country (n, %) |  |  |  |  |
| Uganda | 240 (55) | 127 (54) | 113 (56) | 0.58 |
| South Africa | 198 (45) | 110 (46) | 88 (44) |  |
| Current smoker (n, %) | 50 (11) | 24 (10) | 26 (13) | 0.27 |
| Pre-treatment log10 viral load copies/mL (median, IQR) | 4.5 (3.8 – 5.1) | 4.1 (3.3 – 4.6) | 5.0 (4.5 – 5.4) | <0.001 |
| Pre-treatment CD4 count cells/μL (median, IQR) | 366.5 (132 – 437) | 428 (392 – 472) | 124 (69 – 163) | N/A |
| Soluble CD14  (median, IQR) | 1363 (1108 – 1711) | 1203 (1024 – 1465) | 1598 (1290 – 2038) | <0.001 |
| Interluekin-6  (median, IQR) | 0.84 (0.53 – 1.67) | 0.67 (0.41 – 1.07) | 1.23 (0.72 – 2.81) | <0.001 |
| D-dimer  (median, IQR) | 0.53 (0.27 - 1.06) | 0.43 (0.23 – 0.69) | 0.78 (0.37 – 1.38) | <0.001 |

*P*-value results represent statistical tests of comparison between earlier and late ART sub-group characteristics, performed with rank-sum testing for continuous variables and chi-squared testing for categorical or dichotomous variables.

ART: antiretroviral therapy; IQR: interquartile range

Supplemental Table 2: Association between change in biomarkers of inflammation from pre-treatment to 12 months and earlier/asymptomatic versus late-stage disease at antiretroviral therapy initiation, with and without adjustment for confounding factors, including individuals unsuppressed at 12 months (n=551)

|  | **Soluble CD14** | | | | **IL-6** | | | | **D-Dimer** | | | |
| --- | --- | --- | --- | --- | --- | --- | --- | --- | --- | --- | --- | --- |
|  | Univariable | | Multivariable | | Univariable | | Multivariable | | Univariable | | Multivariable | |
|  | Estimate (95%CI) | *P*-value | Estimate (95%CI) | *P*-value | Estimate (95%CI) | *P*-value | Estimate (95%CI) | *P*-value | Estimate (95%CI) | *P*-value | Estimate (95%CI) | *P*-value |
| Age (10 yrs.) | 0.03  (-0.04, 0.10) | 0.44 | 0.01  (-0.05, 0.08) | 0.66 | **0.02**  **(-0.05, 0.10)** | **0.05** | 0.04  (-0.03, 0.0.12) | 0.27 | **0.09**  **(0.02, 0.17)** | **0.01** | **0.10**  **(0.02, 0.17)** | **0.01** |
| Female Sex | **0.14**  **(-0.28, -0.0006)** | **0.05** | -0.05  (-0.18, 0.09) | 0.52 | -0.12  (-0.27, 0.04) | 0.13 | 0.04  (-0.12, 0.20) | 0.64 | **-0.18**  **(-0.33, -0.02)** | **0.02** | -0.04  (-0.19, 0.12) | 0.65 |
| South Africa (vs Uganda) | **0.14**  **(0.004, 0.28)** | **0.04** | 0.13  (-0.01, 0.26) | 0.06 | **-0.22**  **(0.38, -0.73)** | **0.004** | **-0.28**  **(-0.43, -0.13)** | **<0.001** | -0.03  (-0.18, 0.12) | 0.71 | -0.10  (-0.25, 0.05) | 0.21 |
| Current Smoking | -0.03  (-0.24, 0.18) | 0.79 | -0.14  (-0.36, 0.07) | 0.19 | 0.01  (-0.23, 0.25) | 0.39 | 0.07  (-017, 0.32) | 0.56 | 0.07  (-0.1.7, 0.31) | 0.56 | 0.01  (-0.23, 0.25) | 0.93 |
| Pre-treatment VL (Log10) | **0.22**  **(0.15, 0.29)** | **<0.001** | **0.11**  **(0.04, 0.19)** | **0.002** | **0.17**  **(0.10, 0.25)** | **<0.001** | **0.09**  **(0.00, 0.17)** | **0.04** | **0.02**  **(0.13, 0.28)** | **<0.001** | **0.13**  **(0.05, 0.22)** | **0.002** |
| **Late ART (vs Earlier ART)** | **0.26**  **(0.20, 0.33)** | **<0.001** | **0.42**  **(0.27, 0.56)** | **<0.001** | **0.26**  **(0.18, 0.33)** | **<0.001** | **0.43**  **(0.27, 0.59)** | **<0.001** | **0.22**  **(0.15, 0.29)** | **<0.001** | **0.30**  **(0.13, 0.46)** | **<0.001** |

*Biomarkers of inflammation log-transformed and divided by the inter-quartile range of the distribution, such that the coefficient represents a 1-unit change in the interquartile range for that biomarker

ART: Antiretroviral therapy; VL: HIV-1 RNA viral load

Supplemental Table 3: Association between earlier/asymptomatic versus late-stage antiretroviral therapy initiation and biomarkers of inflammation at pre-treatment, 12-months, and change from pre-treatment to 12 months, with and without adjustment for pre-treatment viral load*

|  | **Soluble CD14** | | | | **IL-6** | | | | **D-Dimer** | | | |
| --- | --- | --- | --- | --- | --- | --- | --- | --- | --- | --- | --- | --- |
|  | Adjusted for Pre-treatment Viral Load | | Not Adjusted for Pre-treatment Viral Load | | Adjusted for Pre-treatment Viral Load | | Not Adjusted for Pre-treatment Viral Load | | Adjusted for Pre-treatment Viral Load | | Not Adjusted for Pre-treatment Viral Load | |
| **Late ART (vs Earlier ART)** | Estimate (95%CI) | *P*-value | Estimate (95%CI) | *P*-value | Estimate (95%CI) | *P*-value | Estimate (95%CI) | *P*-value | Estimate (95%CI) | *P*-value | Estimate (95%CI) | *P*-value |
| Pre-treatment | **0.53**  **(0.40, 0.66)** | **<0.001** | **0.74**  **(0.60, 0.87)** | **<0.001** | **1.67**  **(1.27, 2.06)** | **<0.001** | **2.14**  **(1.78, 2.51)** | **<0.001** | **1.30**  **(0.90, 1.70)** | **<0.001** | **1.85**  **(1.48, 2.22)** | **<0.001** |
| 12 months after ART | -0.00  (-0.17, 0.17) | 0.99 | 0.10  (-0.05, 0.26) | 0.19 | 0.05  (-0.33, 0.43) | 0.80 | 0.32  (-0.02, 0.66) | 0.07 | 0.10  (-0.36, 0.57) | 0.66 | 0.33  (-0.09, 0.75) | 0.12 |
| Change from pre-treatment to 12 months after ART | **0.43**  **(0.27, 0.59)** | **<0.001** | **0.53**  **(0.40, 0.68)** | **<0.001** | **0.44**  **(0.26, 0.61)** | **<0.001** | **0.51**  **(0.35, 0.67)** | **<0.001** | **0.28**  **(0.10, 0.46)** | **0.003** | **0.39**  **(0.22, 0.55)** | **0.001** |

*Models adjusted for age, sex, current smoking status, and country of origin. Biomarkers of inflammation log-transformed and divided by the inter-quartile range of the distribution, such that the coefficient represents a 1-unit change in the interquartile range for that biomarker

ART: Antiretroviral therapy; VL: HIV-1 RNA viral load
